# Toxic Elemental Impurities in Herbal Weight Loss Supplements; A Study Using ICP-OES Microwave-Assisted Digestion

**DOI:** 10.3390/toxics11030272

**Published:** 2023-03-16

**Authors:** Ghanim Al-Thani, Adel Ehab Ibrahim, Mohammed Alomairi, Baher I. Salman, Mostafa M. Hegazy, Ahmed Al-Harrasi, Sami El Deeb

**Affiliations:** 1 Natural and Medical Sciences Research Center, University of Nizwa, PC 616, Birkat Al Mauz, Nizwa P.O. Box 33, Oman; 2 Pharmaceutical Analytical Chemistry Department, Faculty of Pharmacy, Port-Said University, Port Said 42526, Egypt; 3 Pharmaceutical Analytical Chemistry Department, Faculty of Pharmacy, Al-Azhar University, Assiut Branch, Assiut 71524, Egypt; 4 Department of Pharmacognosy and Medicinal Plants, Faculty of Pharmacy, Al-Azhar University, Cairo 11884, Egypt; 5 Institute of Medicinal and Pharmaceutical Chemistry, Technische Universitaet Braunschweig, 38106 Braunschweig, Germany

**Keywords:** trace elements, dietary supplements, ICP-OES, microwave digestion

## Abstract

The tendency of using weight loss herbal preparations is continuously increasing, especially for the widespread consumption of junk food that is characterized by high calories. Weight loss herbal preparations are considered a type of food supplement product, and, as such, the regulations governing their quality control might be minimal. These products could be locally formulated in any country or internationally imported. Being non-controlled products, the herbal weight-loss products may contain high levels of elemental impurities that might exceed the permissible ranges. Moreover, these products contribute to the total daily intake (TDI) of such elements, which might represent concerns about their potential toxicological danger. In this research, the elemental contents in such products were investigated. The inductively coupled plasma with optical emission spectrometer (ICP-OES) was used to determine the levels of 15 elemental contents, namely, Na, K, Ca, Mg, Al, Cu, Fe, Li, Mn, As, Co, Cr, Cd, Ni and Pb. The results showed that seven micro-elements, namely Cd, Co, Ni, Cr, Pb, Li and Cu, were either not detectable or at a concentration much lower than their tolerable limits. However, all studied macro-elements (Na, K, Ca and Mg), together with Fe, were found at considerable, yet safe levels. On the other hand, Mn, Al and As contents showed perturbing levels in some of the studied products. Finally, a conclusion was highlighted for the necessity for stricter surveillance of such herbal products.

## 1. Introduction

Food supplements, also referred to as dietary supplements, is a big sector in Omani market that is acquiring importance. There is a continuous increase in the consumption of food supplement products. Consumers are usually seeking the high-quality and safe products among popular brands. Dietary supplements can be classified into two subclasses or categories, namely herbal preparations and minerals and vitamins [1,2]. In term of official regulation of these products, the European countries and the United States of America place dietary supplement in a grey zone between nutrients and drugs. That is to say, the quality control measures required for the registration of a dietary supplement are not as critical and strict as that for drugs labeled as non-medicinal products but also are more than that required for some food products. Unfortunately, some dietary supplement preparations might escape regulatory and registration conditions and reach the market. They are regarded as food grade and, thus, are not regulated as drugs [3,4].

The consumption of dietary products might seem safe to the consumer, but it has been sometimes correlated with diseases such as liver injury [5]. In this regard, it is worth noting that variation in cultivation region, climatic conditions, type of the soil, containments in soil, water and air or even intentional adulteration can result in different impurities in the claimed plant products [6,7,8,9,10,11]. For instance, herbal cultivated in areas near industries or heavy traffics can accumulate toxic elements such as arsenic and lead [12]. The contamination of herbs with heavy metals is considered a health threat [12]. It is also worth mentioning that cultivated herbs are more favorable than wild-harvested herbs for the preparation of herbal products by pharmaceutical companies because it shows less variation in constituents [13].

Element profiling is an important issue for safety. Using a major element classification, levels of main macro-elements (e.g., Na, K, P, Mg, Ca) and essential micro-elements (e.g., Cu, Mn, Fe, Zn) should not exceed the recommended daily allowances (RDAs) and should be quantified to avoid toxicity if consumed in excess. Trace elements are essential for humans only in trace amounts and will be toxic at supra optimal levels [14,15]. Moreover, the possible presence of toxic elements even in low levels (e.g., As, Cd, Cr, Ni and Pb) in these products should carefully be tested to ensure safety [16,17].

There is a large volume of published studies about the determination of the elemental impurities content in herbal medicines. One such study was conducted by a German working group who ran a heavy metals database for over 12,000 samples corresponding to 204 herbal drugs and tested for lead and cadmium content [18]. There are some published strong evidences that show that the herbal medicines from Asia contain toxic metals [19], including the traditional Chinese medicines, and these studies detected a high concentration of arsenic and mercury in certain herbal-ball preparations [20]. Another study of traditional Chinese medicine was performed in Singapore and found that 93 cases of excessive toxic heavy metals and undeclared drugs were detected [21]. According to one of the published research articles in 2004, the second Asian country that has a rich history of herbal medicine is India, and one of its products is called Ayurvedic Herbal Medicine (AHM), which was found to contain high levels of lead, mercury, and arsenic [22].

Nutritional supplements are classified differently in different countries. For instance, in the Sultanate of Oman, there are two primary parts which are determined by the information on the label. If the products are labeled for food use, they will be regulated by the Food, Safety and Quality Center. However, if the supplements are labeled for medicinal use, they will be regulated by the Ministry of Health’s Directorate General of Pharmaceutical Affaires and Drug Control. To the best of our knowledge, there is no reported study on the investigation of elemental impurities in weight-loss products available in the Omani market. The check for potential elemental impurities will help in improving the health status in this sector to reveal the potential health impact of using metal contaminated herbal products with over-limit main and/or essential elements or heavy metals. The aim is to get clear picture of the elemental composition of weight loss products. Investigating the presence of macro-elements, micro-elements and/or possible toxic elements as impurities in different herbal weight loss products will test whether they exceed the allowed values and, thus, can represent a possible health risk. Studied products will be classified in terms of potential metal toxicity based on the elemental profiling results.

## 2. Experimental

### 2.1. Instrumentation & Reagents

All samples were analyzed using the Optima 8000 ICP-OES system from Perkin-Elmer (Waltham, MA, USA). The instrument was equipped with a Meinhard nebulizer and cyclonic glass spraying chamber. This equipment uses a dual backside-illuminated, charge-coupled device detector type, with wavelength range 160–900 nm. Data were handled using Syngistix^®^ software (v 2.3) for ICP-OES by Perkin-Elmer (Waltham, MA, USA).

Samples were digested using an ultraWAVE^®^ microwave digestion system from Milestone (Sorisole, Italy). All used tools and containers were washed carefully using ultrapure water (conductivity ≈ 0.4 µS cm^−1^) and then 20% nitric acid before experimentation.

Nitric acid (65%, *w/v*) and hydrogen peroxide (30%, *w*/*v*) were of analytical grades and were purchased from Merck (Darmstadt, Germany). For calibration and validation of the proposed methodology, a standard solution containing the targeted multi-elements was purchased from Merck (Darmstadt, Germany) and was at a concentration of 1000 mg L^−1^. Ultrapure water was used (conductivity ≈ 0.4 µS cm^−1^) during the study which was generated by a Millipore water purification system (Burlington, MA, USA).

### 2.2. Sample Collection and Preparation

A total of 18 different samples of weight loss food supplements were purchased from the Omani market. The following details were recorded for each sample; formula of the product, product country and recommended daily dosage (Table 1).

The samples were first homogenized before starting the analysis procedure. About 0.5 g of each homogenized sample was digested in the microwave decomposition apparatus that was equipped with temperature and pressure control by the addition of 3 mL nitric acid and 1.5 mL hydrogen peroxide into an acid-washed, modified polytetrafluoroethylene (TFM) digestion tube [23]. About 120 mL of water were inserted into the single reaction chamber (SRC) and pressurized up to 40 bar in order to keep the TFM tube caps in the upper part of the quartz vessel. The samples were then transferred to the microwave digestion with condition lines shown in Table 2. The chamber was left until the samples cooled down then it was depressurized. The sample were obtained as solutions containing their digests, which were then diluted to 50mL volume in polypropylene flasks using 0.5M nitric acid in order to be analyzed using ICP-OES [24].

### 2.3. ICP-OES Methodology

The conditions of ICP-OES had been optimized for better measurement sensitivities. The optimum conditions are listed in Table 2. Plasma argon gas was set at 8.0 L min^−1^ flow rate, and auxiliary gas at 0.2 L min^−1^. The pump flow rate was set at 1.5 mL min^−1^.

The emission lines for the studied elements were chosen from the previous laboratory studies that showed minimal interferences. The chosen emission lines for micro-elements were as follow; As 193.696, Cd 228.802, Co 228.616, Ni 231.604, Cr 267.716, Mn 257.610, Pb 220.353, Li 670.784, Cu 327.393, Fe 238.204 and Al 396.153 for micro-elements, while emission lines used for macro elements were Na 589.592, K 766.490, Mg 285.213 and Ca 317.933. The emission view for micro-elements was axial enhanced sensitivity, while for macro-elements it was radial for better specificity and selectivity.

### 2.4. Standard Preparation

The working standards were prepared from the multi-element standard solution with dilution in 0.5 M nitric acid. For construction of calibration curves, five working standards were prepared at concentrations 0.1, 1.0, 5.0, 7.0 and 10.0 mg L^−1^ for the micro-elements (As, Cd, Co, Ni, Cr, Mn, Pb, Li, Cu, Fe and Al). Another six linearity working standards at concentrations 1.0, 25.0, 50.0, 100.0, 150.0 and 200.0 mg L^−1^ were prepared for the macro-element in the same manner (Na, K, Mg and Ca).

For ensuring the accuracy and precision validation parameters and in order to ensure recoveries due to digestion procedure, three spiked quality control (QC) standards were prepared using the same dosage forms under study. 100 mg of each chosen sample was spiked with micro-elements at concentrations 0.5, 5.0 and 10.0 mg L^−1^, while for macro-elements the QC standards concentrations were at concentrations 5.0, 50.0 and 150.0 mg L^−1^.

### 2.5. Analytical Method’s Performance and Validation

The method was validated using the ICH guidelines for testing the accuracy, precision and matrix effects for spectroscopic analytical techniques [25,26]. Linearity was established across the validation ranges which were 0.1–10.0 mg L^−1^ for micro-elements (As, Cd, Co, Ni, Cr, Mn, Pb, Li, Cu, Fe and Al) and 1.0–200.0 mg L^−1^ for macro-elements (Na, K, Mg and Ca). Replicate injections of the QC standards were conducted in order to calculate recovery percentages and confirm the method’s accuracy. The precision was established in the form of repeatability and intermediate precision of the obtained recovery percentages of the QC standards. Blank experiments using the three chosen dosage forms were performed for subtracting the background signal intensities. Each QC standard was injected in triplicate as well as its corresponding blank.

ICP-OES constitutes a good analytical technique for the identification and quantitation of elements in different products with high accuracy and precision using ICP multi-element standards and multi-wavelength calibration. A sample preparation to digest the product using microwave in acidic media, also referred to as microwave assisted mineralization or microwave assisted digestion, is usually advantageous in sample preparation [27]. Time saving is one of the primary advantages of microwave digestion where up to 12 samples can be prepared in the same cycle which lasts as little as 10 min. The environmental safety represented by the low acid consumption and operator safety indicated by the absence of exposure to acidic fumes are other advantages over the open-air digestion technique. Finally, the chance for loss of volatile elements, such as Pb or sample contamination, are minimal in microwave digestion technique.

The linearity of the proposed method was checked within the specified ranges for micro- and macro-elements. The obtained results of coefficients of variation calculated for each element as shown in Table 3 indicate good linearity. The accuracy of the used methodology was assessed by calculating the percentage recoveries of the QC standards after being digested in the same manner for the samples. The calculated mean values for recovery percentages are shown in Table 3. The recovery results for the QC standards confirm the closeness of true and found elemental concentrations. For precision study, the repeatability was assessed by injecting six replicates of one QC standard at concentration 5.0 mg L^−1^ for micro-elements and at concentration 50.0 mg L^−1^ for macro-elements and then calculating the standard deviation (SD) of the obtained results. For intermediate precision, the same QC standard was injected on three different days in duplicates; then the standard deviation (SD) of the results was calculated. Table 3 shows the results of the repeatability and intermediate precision standard deviations which indicate the close agreements of the series of measurements.

## 3. Results and Discussion

### Determination of Elemental Contents in Dietary Herbal Supplements

The so-called herbal pharmaceuticals, herbal medicine, herbal drugs or medicinal plants include the plants used to treat, cure or prevent diseases or enhance general health. They are used as herbal preparations. The WHO provided guidelines for the standardization of herbal drugs [28]. According to the WHO, the dependence on nonconventional medicine, mostly herbal, represents 70–80% of the world’s population [29]. Even though those herbal preparations can act as a source of essential elements to the body, possible uncontrolled use of these preparations for, example weight loss products, may cause a health risk and should not be underestimated.

The WHO also indicates that the non-rational use of these products could result in several health problems and chronic diseases. A number of human health disorders, including brain, kidney, liver, pancreas, bone marrow, reproductive system and nervous system disorders, have been attributed to elemental accumulation in the body depending on solubility and bioavailability. Heavy metals are characterized by having low excretion in human [16,30,31,32,33,34,35,36,37]. Cases of human poisoning due to the use of traditional herbal preparations have been reported [38,39]. In Brazil, the levels of elemental impurities present in medicinal plants showed concentrations above that allowed for As, Cr, Cu, Ni and Pb and even higher concentrations for As, when compared to the maximum limits allowed by the Brazilian Pharmaceutical authority [32]. In another study conducted in the Jordanian market, their results showed acceptable toxic metal levels in the finished pharmaceutical products and the traditional medicinal herbs for infants, but they concluded that the use of medicinal plants as alternative medicine should be used with caution, keeping in mind the safety factor for infants [40]. In the UAE, a cross-sectional study for heavy metals in supplemental food was conducted, and the results showed low levels of metals in the products that were available for sale in Dubai, with few exceptions [41].

In most cases, the elementary content of herbal products is not specified; however, specified investigations should ensure the agreement between the measured and certified concentration. Therefore, elemental profiling is useful to improve the quality of nutritional products [42]. For instance, the USP elemental impurities limits described methods for the determination of elemental impurities using ICP-OES (procedure 1) [43]. In fact, adherence to quality control regulatory guidelines of herbal medicines to test quality, safety and efficacy may differ in different countries depending on cultural aspects. Instead of a global regulatory mechanism, several local regulatory models for herbal medicines (over-the-counter drugs, traditional medicines, dietary supplements, prescription drugs) are found [13]. Moreover, the legislation regulating the limits of metal impurities even differ between international authorities [44]. Table 4 shows the concentration limits and permissible daily exposure dose for some elemental impurities based on the European Medical Agency (EMA), the United States pharmacopeia (USP) [44,45,46,47]. However, owing to the absence of legislative supervision, the current situation puts the safety of these dietary food supplements to be exclusively ensured by the producer to ensure authenticity and health safety.

To our knowledge, no studies were performed on the herbal weight loss preparation found in the Omani market. Therefore, this research study would be the first to be conducted. Table 5 shows the results obtained for screening 15 elements in the selected herbal weight loss preparations under study. The results are given in µg per gram of the powdered substances for the purpose of comparison to the concentration limits obtained from different sources and the literature (Table 4). All samples showed considerable amounts of the macro-elements (Ca, Mg, K, Na), together with Al, Fe and Mn from the micro-elemental content. This, in turn, alarms for the importance of labeling the amounts of each of these elements.

Seven micro-elements from the studied group were not considerably found in almost all the samples. Four metals including Pb, Co, Ni and Cd were almost not detectable in all the studied samples. The other three elements of Cu, Cr and Li were found in some herbal weight loss samples but at low concentrations, much below their toxic limits (Table 5). A Cr absence was not expected, although several research papers describe its beneficial influence on attenuating the weight gain and improving insulin sensitivity [48,49]. On the other hand, As, Fe, Al and Mn were detectable in all samples (Table 5). Moreover, the risks could be higher when calculating the daily ingested elemental intake from the studied herbal weight loss preparations as prescribed in the labeled doses (Table 1). The daily intake was calculated for each element (Table 6) according to the labeled usage of each product which was prescribed to be three times of each dosage form before or after meals. As shown, Cr, Cu and Li were all below the PDE (Table 4). Table 6 calculates all the other elemental daily exposure doses, assuming the average labeled use of each product is three times daily.

Macro-elements are essential for body functions when consumed within certain amounts. Elements such as Na, K, Ca and Mg are beneficial for maintaining the body’s homeostasis and maintaining body skeleton structure, besides other metabolic functions [50,51,52]. However, their over intake could cause several disorders. A maximum daily intake of Na without risk exposure was reported to be 2500 mg per day [53]. High daily Na intake strongly provokes cardiovascular disorders such as elevated blood pressure. Safe daily intake of K, Ca and Mg can be up to 7400, 1100 and 800 mg daily [53]. Therefore, according to those reported limits (as shown in Table 6), the studied herbal products are within acceptable daily intake thresholds. Therefore, the importance of labeling elemental content applies for all macro-elements, especially in abnormal human body conditions (e.g., hypertension for ingested sodium intakes).

Moreover, Mn is an essential element for humans, but its over-exposure can lead to neurological disorders [54], fatigue and sexual dysfunctions [55]. Some studies reported its maximum recommended daily dose for adults to be within the range of 3.5–7.0 mg/day [56]. As shown in Table 6, one of the studied products (A17) showed a high level of Mn content that exceeded its maximum recommended daily dose [56].

For iron daily exposure, the elemental iron supplements for pregnant women was reported to be within ranges from 30 mg/day up to as high as 240 mg/day in the United States [57]. This daily supplement is even lower, 60–120 mg/day according to the International Nutritional Anemia Consultative Group (INACG) [57]. As shown in Table 6, the USA limit was not exceeded for the products under study. Another recent research exploited the etiology of severe iron toxicity was through ingesting more than 60 mg/kg body weight per day [58]. The cited report demonstrated moderate to severe toxicity at even lower daily intakes. The maximum tolerable daily intake (MTDI) of iron was reported as 0.8 mg/day/Kg body weight [59]; therefore, an average adult weight of 70 Kg can tolerate up to 56 mg iron daily. Assuming the average use of each product three times daily, all iron contents will not exceed the MTDI.

The MTDI of elements is sometimes calculated as dose per kg of human body weight. A study was conducted to estimate the average adult weights in Asia, Europe, Africa, Latin America, Northern America and Oceania [60], and this was used to calculate an average of about 70 kg for adult weight. Therefore, calculating the PDE for an average adult in the proposed research was built on that average (70 kg). As an example, As was reported to have a daily tolerable intake of about 2.1 µg/kg body weight per day [12,61], which corresponds to 147 µg As/day. As shown in Table 6, considerable amounts of As were found in some of the studied herbal weight loss products, of which two products exceeded the MTDI (products A6 and A7) and one product was near the threshold (A17). Arsenicosis (As toxicity) is taken seriously in consideration for serious illness without effective therapeutic measures and could lead to irreversible damage to bodily organs and carcinogenicity [62]. Knowing that As is considered the twentieth most plentiful element within the earth sphere [62] and taking that together with the acquired results, it must be logical to control its limits in any human ingestible food or drug.

Although Al was previously considered to be safe, several research papers questioned its relation to Alzheimer’s disease, breast cancer and neurologic renal failure [63]. Al is widely distributed element on the earth surface and has low bioavailability [64]. The MTDI from Al, for example, was reported to be 0.00164 mg/kg body weight per day [65] and also was reported to be 2 mg/kg body weight per week by The Joint Food and Agricultural Organization/WHO Expert Committee on Food Additives (JECFA) [64]. Either limit could be used; nevertheless, these limits are threatened by some of the weight loss products under study, as shown in Table 6. Using an average of 70 Kg for adult body weight as mentioned earlier, this limit would be calculated as 114 µg Al/daily or 140 mg Al/ weekly. As calculated in Table 6, almost all the studied herbal weight loss products’ daily intake, except two products (A7 and A18), exceeded the MTDI of Al.

## 4. Conclusions

An analytical ICP-OES methodology was developed for determination of macro- and micro-elements in herbal products. A sample of eighteen different herbal weight loss products marketed in Oman were investigated for their elemental contents. The samples showed diversity in their elemental contents—either macro-elements, essential and/or toxic elements. Four elements were undetectable including Pb, Co, Ni and Cd. Eight elements encompassing all macro-elements, Na, K, Ca and Mg, together with Fe, Cu, Cr and Li micro-elements, were found within safe daily exposure limits. However, three elements were found above their maximum daily tolerable limits in some products; of which, Mn was found in one herbal weight loss product. Al and As were counted in all products; of which, 2 products were alarming for As and 14 products for Al. The discrepancies in the studied products’ elemental contents sound an alarm for the unreliability of their quality appraisal. Therefore, stricter measures on the quality control of such herbal products are important for improving the consumers’ health. The proposed study is the first to be conducted in Oman which could be beneficial in improving the health status in this herbal market sector by signaling the potential elemental impurities.

## Figures and Tables

**Table 1:** List of weight loss products under study.

Product Serial	Formula	Labeled Origin Country	Weight (g) per Dose
A1	Sachets	Turkey	8.5 g
A2	Tablet	Yemen	0.7 g
A3	Powder	Turkey	2.0 g *
A4	Powder	Oman	1.7 g *
A5	Teat sachets	kuwait	2.7 g
A6	Coffee sachets	Not labeled	15.0 g
A7	Coffee sachets	Thailand	15.0 g
A8	Tea powder	Turkey	5.0 g *
A9	Tablet	USA	0.8 g
A10	Herbal mix sachets	Egypt	1.8 g
A11	Powder	Egypt	2.6 g
A12	Packets	Egypt	1.8 g
A13	Sachets	Egypt	1.2 g
A14	Herbal mix packets	Egypt	2.5 g
A15	Herbal-mix powder	Oman	1.4 g *
A16	Tablets	Russia	4.0 g
A17	Coffee powder	Turkey	10.0 g *
A18	Tea powder	Iran	2.8 g *

* Weight per one dosing spoonful as indicated by the product leaflet.

**Table 2:** Microwave oven and ICP-OES condition lines.

Microwave Digestion Conditions				
Step	Power	Time	Temperature	Pressure
1	800 W	10 min	110 °C	80 bar
2	1000 W	10 min	180 °C	80 bar
3	1500 W	10 min	240 °C	120 bar
**ICP-OES**				
Rf power (W)			1500	
Injector			Alumina 2 mm I.D. *	
Sample tubing			Standard 1.14 mm I.D. *	
Drain tubing			Standard 1.14 mm	
Quartz torch			Single slot	
Sample capillary			PTFE 1.0 mm internal diameter	
Sample vials			Polypropylene	
Source equilibrium delay			15 s	
Plasma viewing			Axial for micro-elements; Radial for macro-elements	
Processing mode			Peak area	
Plasma gas			Argon	
Nebulizer gas flow			0.7 L min^−1^	
Shear Gas			Air	

* I.D., Internal Diameter.

**Table 3:** Validation data for the determination of elements in herbal weight loss preparations.

Element	R^2^	Repeatability **	Inter-day Precision **	Accuracy ***
MicE *		50.0 mg L^−1^		Recovery% ± SD
As	0.999	0.1	3.5	93.4 ± 0.1
Cd	0.999	0.1	1.7	100.8 ± 0.1
Co	0.999	0.2	0.6	100.7 ± 0.1
Ni	0.999	0.1	1.4	102.6 ± 0.2
Cr	0.999	0.1	3.0	103.2 ± 0.1
Mn	0.999	0.3	2.7	107.2 ± 0.1
Pb	0.999	0.1	0.7	99.8 ± 0.2
Li	0.999	0.1	1.1	85.7 ± 0.3
Cu	0.999	0.1	2.7	106.5 ± 0.1
Fe	0.999	0.1	3.1	90.1 ± 0.1
Al	0.999	0.1	2.8	111.2 ± 0.1
MacE *				
Mg	0.999	0.2	1.3	116.1 ± 0.3
K	0.999	0.1	2.6	115.0 ± 0.4
Na	0.999	0.5	3.7	87.1 ± 0.1
Ca	0.999	0.1	2.2	112.8 ± 0.1

* MicE, micro-elements; MacE, macro-elements; ** Data of standard deviation results (n = 6); *** Data of Recovery% ± standard deviation (*n* = 9).

**Table 4:** Elemental impurity limits according to current EMA, WHO and USP guidelines.

Element	Concentration Limit (µg/g)	PDE * (µg/day)
Cd	0.5	5
Co	5.0	50
Ni	20.0	200
Cr	1100.0	11,000
Pb	1.5	5
Li	55.0	550
Cu	300.0	3000

* Permissible daily exposure.

**Table 5:** Elemental impurities measured in 18 herbal weight loss samples.

Sample	As	Cd	Co	Ni	Cr	Mn	Pb	Li	Cu	Fe	Al	Na	K	Ca	Mg
	MicE * (µg/g)											MacE * mg/g			
A1	3	nd **	nd	nd	nd	9	nd	nd	nd	56	45	0.64	2.17	0.26	6.38
A2	10	nd	1	nd	17	65	nd	1	8	1438	1297	1.56	7.02	0.60	7.94
A3	4	nd	nd	nd	nd	118	nd	nd	nd	403	283	0.81	6.14	0.20	3.65
A4	1	nd	nd	nd	nd	115	nd	1	3	388	277	2.24	10.42	0.87	7.98
A5	3	nd	nd	nd	nd	50	nd	nd	11	502	585	3.10	13.13	1.87	8.40
A6	5	nd	nd	nd	23	3	nd	nd	nd	13	6	0.35	8.99	1.54	0.41
A7	4	nd	nd	nd	nd	2	nd	nd	nd	9	1	0.26	7.09	1.25	0.37
A8	4	nd	nd	nd	nd	8	nd	nd	nd	32	18	0.52	2.01	0.48	2.75
A9	5	nd	nd	nd	nd	25	nd	nd	nd	59	73	0.43	0.25	0.37	36.28
A10	4	nd	nd	3	nd	22	nd	nd	8	37	2	1.45	6.01	0.63	3.35
A11	4	nd	nd	4	nd	632	nd	nd	11	351	951	1.33	10.50	0.00	2.91
A12	4	nd	nd	3	nd	629	nd	nd	10	615	1259	2.30	12.35	1.36	8.76
A13	3	nd	1	nd	nd	196	nd	15	nd	457	532	4.85	14.88	0.46	9.84
A14	4	nd	nd	nd	nd	35	nd	1	8	456	666	2.45	13.58	0.77	11.30
A15	3	nd	nd	nd	11	67	nd	2	10	1080	1318	3.18	16.46	2.49	10.44
A16	4	nd	nd	nd	nd	0	nd	nd	nd	50	149	0.98	0.30	53.66	0.08
A17	4	nd	nd	3	nd	1410	nd	nd	10	97	1065	1.36	9.65	0.14	3.59
A18	4	nd	nd	nd	nd	8	nd	nd	2	25	11	0.47	7.76	1.27	0.38

* MicE, micro-elements; MacE, macro-elements; ** nd; not detectable (below ICP method’s limits of quantitation ≤ 0.009 mg L^−1^).

**Table 6:** The cumulative daily exposure dose for each element studied in the herbal weight loss samples.

Sample	As	Cr	Mn	Li	Cu	Fe	Al	Na	K	Ca	Mg
	µg/day							mg/day			
A1	76.5	0.0	229.5	0.0	0.0	1428.0	1147.5	16.3	55.3	6.6	162.7
A2	21.0	35.7	136.5	2.1	16.8	3019.8	2723.7	3.3	14.7	1.3	16.7
A3	24.0	0.0	708.0	0.0	0.0	2418.0	1698.0	4.9	36.8	1.2	21.9
A4	5.1	0.0	586.5	5.1	15.3	1978.8	1412.7	11.4	53.1	4.4	40.7
A5	24.3	0.0	405.0	0.0	89.1	4066.2	4738.5	25.1	106.4	15.1	68.0
A6	225.0	1035.0	135.0	0.0	0.0	585.0	270.0	15.8	404.6	69.3	18.5
A7	180.0	0.0	90.0	0.0	0.0	405.0	45.0	11.7	319.1	56.3	16.7
A8	60.0	0.0	120.0	0.0	0.0	480.0	270.0	7.8	30.2	7.2	41.3
A9	12.0	0.0	60.0	0.0	0.0	141.6	175.2	1.0	0.6	0.9	87.1
A10	21.6	0.0	118.8	0.0	43.2	199.8	10.8	7.8	32.5	3.4	18.1
A11	31.2	0.0	4929.6	0.0	85.8	2737.8	7417.8	10.4	81.9	0.0	22.7
A12	21.6	0.0	3396.6	0.0	54.0	3321.0	6798.6	12.4	66.7	7.3	47.3
A13	10.8	0.0	705.6	54.0	0.0	1645.2	1915.2	17.5	53.6	1.7	35.4
A14	30.0	0.0	262.5	7.5	60.0	3420.0	4995.0	18.4	101.9	5.8	84.8
A15	12.6	46.2	281.4	8.4	42.0	4536.0	5535.6	13.4	69.1	10.5	43.8
A16	48.0	0.0	0.0	0.0	0.0	600.0	1788.0	11.8	3.6	643.9	1.0
A17	120.0	0.0	42,300.0	0.0	300.0	2910.0	31,950.0	40.8	289.5	4.2	107.7
A18	33.6	0.0	67.2	0.0	16.8	210.0	92.4	3.9	65.2	10.7	3.2

## Data Availability

All data are available upon reasonable request from the corresponding author.
